# Microbiological contamination of domestic kitchen sponges and associated hygiene practices

**DOI:** 10.3389/fmicb.2026.1852521

**Published:** 2026-06-30

**Authors:** Assunta De Lella, Rita Nappi, Antonietta Anzalone, Morena Nappa, Roberta Brunetti, Angela Michela Immacolata Montone, Alessandra Esposito, Francesca Garofalo

**Affiliations:** 1 Department of Food Safety Coordination, Istituto Zooprofilattico Sperimentale Del Mezzogiorno, Via Salute, Portici, Italy; 2 Epidemiology and Biostatistics Coordination Department, Istituto Zooprofilattico Sperimentale Del Mezzogiorno, Via Salute, Portici, Italy

**Keywords:** food safety, food-handling environments, Kitchen sponges, Microbial ecosystems, Pseudomonas

## Abstract

**Introduction:**

Kitchen sponges may act as reservoirs of microorganisms in domestic environments and could represent a potential hygiene risk.

**Methods:**

This study examined microbiological contamination in 98 domestic kitchen sponges. Samples were analyzed for *Escherichia coli*, coagulase-positive staphylococci, Enterobacteriaceae, yeasts, molds, and *Pseudomonas* spp. Sponge condition was classified as “acceptable” or “to be replaced,” and participants completed a questionnaire on usage and hygiene practices.

**Results:**

High and heterogeneous microbial contamination was observed, with particularly high loads of Enterobacteriaceae and *Pseudomonas* spp. in some samples. No significant association was found between sponge condition and microbial contamination. Periodic sponge sanitization showed a non-significant trend toward a lower likelihood of sponges being classified as “to be replaced” (OR = 0.39; *p* = 0.06), after accounting for missing responses.

**Discussion:**

Visual inspection was not a reliable indicator of microbiological contamination. Although periodic sanitization may be associated with better macroscopic sponge condition, the relationship was not statistically significant in this study after accounting for missing responses.

## 1 Introduction

In the domestic environment, food safety is strongly influenced by hygiene practices and by the microbiological status of tools and surfaces used during food preparation. Among these, kitchen sponges represent a peculiar and often underestimated item. Despite their widespread use for washing dishes, cookware and utensils, sponges are characterized by a porous structure and high-water retention capacity, which may favor the accumulation and persistence of microorganisms (Mataragka et al., 2025). Several italian studies have highlighted that domestic kitchens may act as critical points for microbial contamination, particularly when cleaning tools are inadequately managed (Catellani et al., 2014; Ferrero et al., 2022; Marotta et al., 2018; Rossi et al., 2013). In this context, kitchen sponges have been described as potential reservoirs of microorganisms due to the combined presence of food residues, organic matter and prolonged moisture, all conditions that support microbial survival and growth (Møretrø et al., 2021b; Mori et al., 2020). The repeated use of the same sponge on different surfaces, including sinks, countertops and food-contact utensils, may further promote the spread of microorganisms within the household, potentially increasing the risk of cross-contamination (Kusumaningrum et al., 2003). This aspect is of particular concern in domestic settings where vulnerable individuals, such as children, elderly people or immunocompromised subjects, are present, as underlined by italian authors focusing on household food safety and consumer behavior (Catellani et al., 2014; Ferrero et al., 2022; Marotta et al., 2018). Previous investigations conducted in Italy and other European countries have reported the presence of both hygiene indicator microorganisms and foodborne pathogens in kitchen sponges, including enterobacteriaceae, coliforms, yeasts and molds, as well as *Escherichia coli, Staphylococcus aureus, Salmonella spp*. and *Listeria monocytogenes* (Alves et al., 2022; Borrusso and Quinlan, 2017). High microbial loads detected on sponges have been associated not only with their intrinsic physical properties, but also with inadequate cleaning, disinfection and replacement practices (Chen et al., 2011). Several sanitation strategies have been proposed to reduce microbial contamination of kitchen sponges, including the use of chemical disinfectants, dishwashers and microwave treatments (Sharma et al., 2009). Studies conducted by research groups have shown that these practices may significantly reduce bacterial loads; however, their effectiveness is strongly influenced by the presence of organic residues and by the frequency of application (Møretrø et al., 2022b, 2021a). Consequently, improper, or irregular sanitation may result in only temporary reductions, allowing rapid microbial regrowth (Moen et al., 2023). The aim of the present study was to investigate the occurrence of foodborne pathogens and food spoilage microorganisms in kitchen sponges collected from domestic households, to assess their potential role in the transmission of foodborne diseases. Furthermore, the relationship between sponge-use habits, hygiene practices and the level of microbial contamination was evaluated, contributing to a better understanding of domestic food safety risks in the Italian household context.

## 2 Materials and methods

### 2.1 Study design and sample collection

This cross-sectional study evaluated the microbiological contamination of domestic kitchen sponges and its association with sponge condition and household hygiene practices. A total of 123 kitchen sponges were distributed to households; of these, 98 sponges were returned to the laboratory in sterile sampling bags and analyzed within 24 h. A structured questionnaire consisting of 13 multiple-choice questions were administered to participants, in accordance with the General Data Protection Regulation (EU 2016/679) and the Italian Data Protection Code (D.Lgs. 196/2003; articles 106 and related deontological rules for scientific research. The questionnaire collected: (i) general information on the household (age of family members, family size and type, presence of vulnerable individuals); (ii) sponge usage, disinfection practices and replacement frequency; and (iii) knowledge related to food safety. Each sponge was subjected to macroscopic evaluation by two independent operators using three qualitative parameters:

(a) degree of wear (1: like new; 2: normally worn; 3: excessively worn);

(b) level of visible dirt (1: low; 2: medium; 3: high);

(c) presence of food debris or extraneous particles (1: 0–2 particles; 2: 3–5 particles; 3: >5 particles).

For each parameter, increasing scores corresponded to poorer sponge condition. The qualitative evaluations recorded in the original study database were subsequently used to classify sponges into two overall categories (“acceptable” and “to be replaced”) for statistical analysis. The categorization was based on expert judgment and interpret the downstream analyses accordingly. Sponges showing moderate-to-severe deterioration in multiple evaluated parameters were generally classified as “to be replaced”. Classification was based on the combined operator assessment of the three evaluated parameters and reflected the overall apparent condition of the sponge. The macroscopic evaluation procedure was performed independently by two operators; however, inter-rater agreement was not formally quantified using statistical agreement measures. This aspect should therefore be considered a methodological limitation of the study.

### 2.2 Microbiological analyses

For quantitative analyses, each sponge was aseptically divided into two portions. A total of 6 g was transferred into buffered peptone water (BPW), while the remaining portion (approximately 4 g) was added to Half Fraser broth for the detection of *Listeria monocytogenes*, according to UNI EN ISO 11290-1:2017, using a 1:10 (w/v) ratio. Samples were homogenized for 60 s at 230 rpm using a peristaltic homogeniser (Stomacher 400 P, Interscience, France). Serial decimal dilutions (1:10) were prepared in BPW (Dilucup). Each sample was then analyzed for the following microbiological determinations: β-glucuronidase-positive *Escherichia coli* count, according to UNI EN ISO 16649-2:2017; Enterobacteriaceae count, according to UNI EN ISO 21528-2:2017; Yeasts and molds count, according to UNI EN ISO 21527-2:2008; Coagulase-positive staphylococci count, according to UNI EN ISO 6888-1:2018; *Pseudomonas spp*. count, according to ISO 13720:2010; *Campylobacter spp*. count, according to UNI EN ISO 10272-2:2017; *Salmonella spp*. detection, according to UNI EN ISO 6579-1:2017; *Listeria monocytogenes* detection, according to UNI EN ISO 11290-1:2017; Anaerobic sulfite-reducing bacteria count, incubated under anaerobic conditions at 37 °C for 24 h, according to UNI EN ISO 15213:2003; *Cronobacter spp*. detection, according to UNI EN ISO 22964:2017. Microbial loads were expressed as colony-forming units (CFU) and categorized into three classes: negative, 1–3 log CFU, and >3 log CFU, in line with previous studies on household hygiene microbiology (Scott et al., 1982; Viana et al., 2025).

### 2.3 Statistical analysis

All statistical analyses were conducted using R software (version 4.2.2). Quantitative descriptive statistics for microbial counts included also non-detect samples, which were treated as zero values for descriptive analyses. Descriptive statistics were used to summarize quantitative variables, including minimum, quartiles, median, mean, and maximum values. The sponge score was dichotomised into *acceptable* and *to be replaced*. Microbiological variables were categorized as described above. Associations between categorical variables were assessed using Fisher's exact test, which is appropriate for small sample sizes. Odds ratios (ORs) and 95% confidence intervals (95% CI) were calculated to estimate the strength of associations. Missing or non-answer responses were treated as missing data and excluded from the corresponding inferential analyses. Given the exploratory nature of the study, the results were interpreted cautiously. A *p*-value < 0.05 was considered statistically significant. No formal sample-size calculation was performed, as the data derived from a broader exploratory research project.

### 2.4 Bacterial colony identification by 16S rDNA sequencing

Genomic DNA was extracted directly from bacterial colonies using the MicroSEQ^®^ ID DNA Extraction Kit (Thermo Fisher Scientific), according to the manufacturer's instructions. Amplification of the 16S rDNA gene was performed using the MicroSEQ^®^ 16S rDNA PCR Kit (Thermo Fisher Scientific). PCR products were checked by agarose gel electrophoresis to confirm successful amplification. Purified amplicons were sequenced using the MicroSEQ^®^ 16S rDNA Sequencing Kit (Thermo Fisher Scientific) on a capillary DNA sequencer (SEQ Studio genetic analyzer- Thermo Fisher Scientific). Sequence analysis and bacterial identification were carried out using the MicroSEQ^®^ ID Analysis Software by comparison with the MicroSEQ^®^ reference database. Identifications were assigned based on sequence similarity, with values ≥99% considered reliable at species level.

## 3 Results

### 3.1 Microbiological analysis and questionnaire evaluation

**Figure 1:**
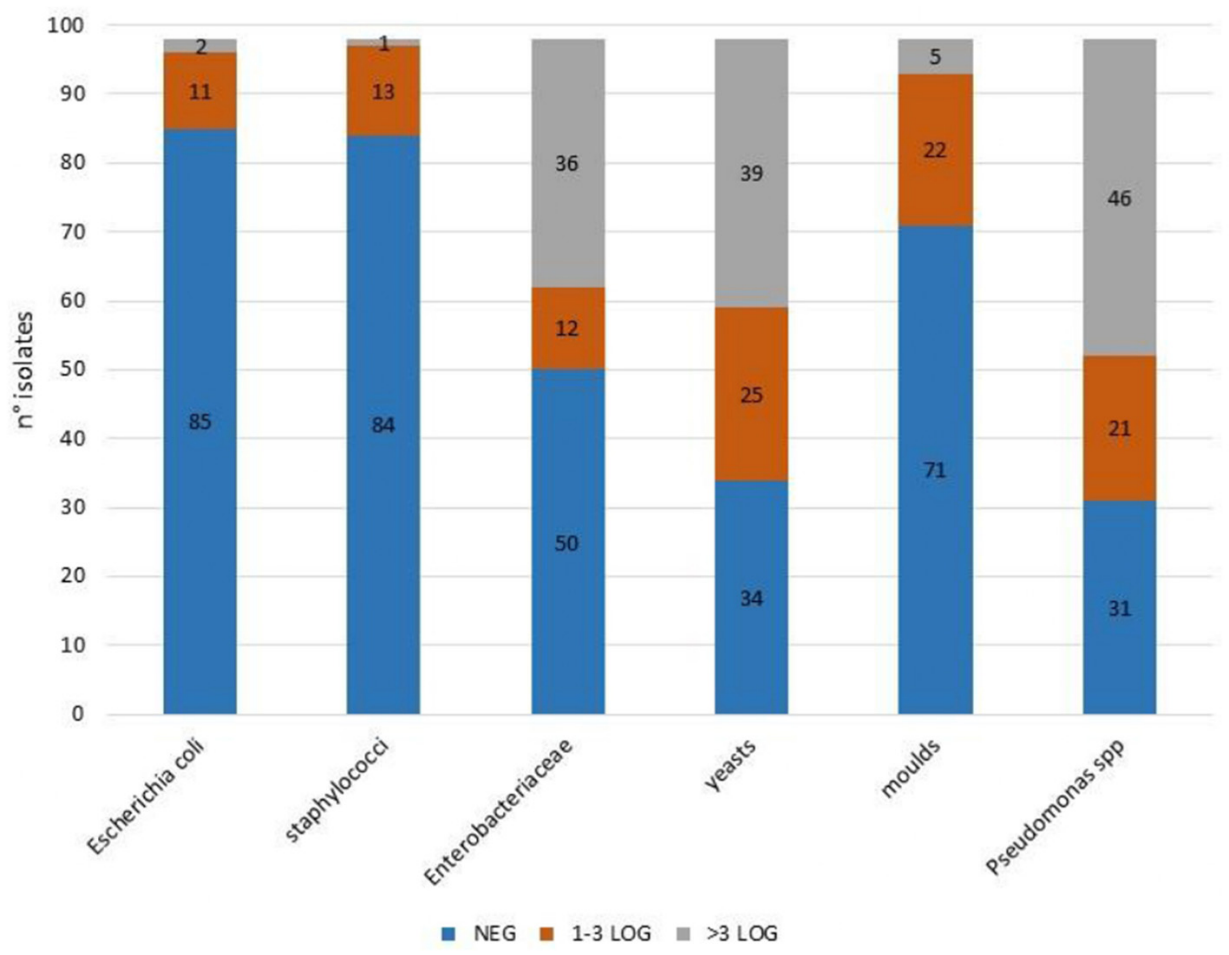
Distribution of microbial contamination levels detected in domestic kitchen sponges. Microbial variables (Escherichia coli, coagulase-positive staphylococci, Enterobacteriaceae, yeasts, moulds, and Pseudomonas spp.) were classified into three contamination categories: negative (non-detect), 1-3 log CFU/sponge, and >3 log CFU/sponge. Values represent the number of sponge samples included in each contamination category.

The microbiological analysis revealed a wide variability in contamination levels across the analyzed kitchen sponges. Results showed a highly heterogeneous distribution of microbial contamination levels among kitchen sponges. Several microbial groups presented median values of 0 log CFU/sponge, indicating a high proportion of non-detect samples, whereas positive samples occasionally reached elevated contamination levels, as reflected by the maximum and upper quartile values. Yeasts and *Pseudomonas* spp. showed comparatively higher median contamination levels than the other investigated microbial groups. (Table 1). These findings indicate the presence of a subset of heavily contaminated sponges alongside relatively clean ones, a pattern consistent with previous reports on domestic hygiene materials (Cardinale et al., 2017; Jacksch et al., 2020). *Campylobacter spp., Salmonella spp., Listeria monocytogenes*, anaerobic sulfite-reducing bacteria, and *Cronobacter spp*. were not detected in any sample. Where the detection limit for *Salmonella spp*. and *Listeria monocytogenes* pathogens is to be considered as total absence in 4 g of sample, while for other microorganisms as < 10 CFU per 6 g of sample. Enterobacteriaceae showed the highest overall contamination, with maximum values reaching 7,88 log_10_, followed by *Pseudomonas* spp. (up to 8.08 log_10_) and yeasts and molds (up to 7.04 log_10_). In contrast, *Escherichia coli* and coagulase-positive staphylococci were less frequently detected at high concentrations, although their presence above 3 log CFU was still observed in a limited number of samples (Figure1). The variable “Sponge Score” has been grouped into only two groups: “acceptable” and “to be replaced”; while, the variables: *Escherichia coli*, staphylococci, Enterobacteria, yeasts, molds, *Pseudomonas* have been grouped into three categories: “NEG”, “1-3 LOG” and “>3 LOG”, Figure 1. No statistically significant associations were observed between sponge score and any of the investigated microbial groups. This applied to *Escherichia coli*, staphylococci, Enterobacteriaceae, yeasts, molds, and *Pseudomonas spp*. (all *p*-values >0.05) (Table 2). Notably, *Pseudomonas spp*. exhibited a trend toward higher contamination in sponges classified as to be replaced, with 58.0% of these sponges showing counts >3 log CFU, compared with 35.4% among acceptable sponges. Although this association did not reach statistical significance (*p* = 0.0568), it may represent an exploratory trend requiring confirmation in larger studies. Questionnaire analysis showed that most participants used kitchen sponges daily (84.7%) and retained the same sponge for more than 1 week (88.8%). More than half of respondents (53.1%) reported that they did not regularly sanitize their sponge, while only 46.9% performed some form of periodic hygienisation. A substantial proportion of households (approximately 43%) included potentially vulnerable individuals, such as elderly people, children under 6 years of age, or pregnant women, highlighting a relevance of domestic sponge hygiene (Table 3). After excluding non-response data from the inferential analysis, a non-significant trend toward an association between periodic sponge sanitisation and sponge condition was observed (OR = 0.39; *p* = 0.06). Sponges from households reporting regular sanitisation were more frequently classified as acceptable; however, this association did not reach statistical significance and should therefore be interpreted cautiously (Table 4). No significant associations were found between sponge condition and food safety knowledge, duration of sponge use, or specific sanitisation methods (Tables 5, 6).

**Table 1:** Descriptive statistics of quantitative variables.

Strains	Min	1°quartile	median	mean	3°quartile	Max
*Escherichia coli*	0.00	0.00	0.00	2.60	0.00	3.18
staphylococci	0.00	0.00	0.00	3.09	0.00	5.00
Enterobacteriaceae	0.00	0.00	0.00	6.35	4.68	7.88
Yeasts	0.00	0.00	3,62	5.56	4.66	7.04
molds	0.00	0.00	0.00	3.59	2.68	5.18
*Pseudomonas* spp	0.00	0.00	3,80	6.65	5.24	8.08

Descriptive statistics of microbial contamination levels detected in domestic kitchen sponges (log CFU/sponge). Non-detect samples were treated as zero values for descriptive statistical analysis. Reported parameters include minimum value (Min), first quartile (Q1), median, arithmetic mean, third quartile (Q3), and maximum value (Max). The first quartile (Q1) represents the value below which 25% of observations fall, while the third quartile (Q3) represents the value below which 75% of observations fall.

**Table 2:** Association between sponge score and microbiological contamination.

CFU/sponge	Acceptable (*N* = 48)	To be replaced (*N* = 50)	*P*-value
***Escherichia coli***			
>3 LOG	2 (4.2%)	0 (0%)	0.414
1–3 LOG	6 (12.5%)	5 (10.0%)	
NEG	40 (83.3%)	45 (90.0%)	
Staphylococci			
>3 LOG	1 (2.1%)	0 (0%)	0.234
1–3 LOG	4 (8.3%)	9 (18.0%)	
NEG	43 (89.6%)	41 (82.0%)	
Enterobacteriaceae			
>3 LOG	16 (33.3%)	20 (40.0%)	0.749
1–3 LOG	6 (12.5%)	6 (12.0%)	
NEG	26 (54.2%)	24 (48.0%)	
Yeasts			
>3 LOG	16 (33.3%)	23 (46.0%)	0.399
1–3 LOG	13 (27.1%)	12 (24.0%)	
NEG	19 (39.6%)	15 (30.0%)	
Molds			
>3 LOG	2 (4.2%)	3 (6.0%)	0.878
1–3 LOG	10 (20.8%)	12 (24.0%)	
NEG	36 (75.0%)	35 (70.0%)	
***Pseudomonas*** **spp**			
>3 LOG	17 (35.4%)	29 (58.0%)	0.0568
1–3 LOG	14 (29.2%)	7 (14.0%)	
NEG	17 (35.4%)	14 (28.0%)	

Association between sponge condition (acceptable vs. to be replaced) and categorical levels of microbial contamination (*Escherichia coli*, coagulase-positive staphylococci, Enterobacteriaceae, yeasts, molds, and *Pseudomonas* spp.). Microbial loads were categorized as negative, 1–3 log CFU, or >3 log CFU. Statistical significance was assessed using Fisher's exact test.

**Table 3:** Frequency distribution of questionnaire responses.

Questionnaire 1	Overall (*N* = 98)
“Fragile subjects” are present in the house	
Seniors	17 (17.3%)
Children < 6 years old	9 (9.2%)
Children < 6 years old—Seniors	2 (2.0%)
Pregnant women	4 (4.1%)
Pregnant women—children < 6 years old	1 (1.0%)
No	56 (57.1%)
Missing	9 (9.2%)
Age Class	
>50	42 (42.9%)
20–30	5 (5.1%)
31–40	19 (19.4%)
41–50	26 (26.5%)
Missing	6 (6.1%)
Gender	
F	52 (53.1%)
M	11 (11.2%)
Missing	35 (35.7%)
Where do you place the kitchen sponge after use	
Sponge holder grid	16 (16.3%)
In a tray	34 (34.7%)
On the sink	41 (41.8%)
Missing	7 (7.1%)
How you wash or clean your dishes	
By hand with the sponge	54 (55.1%)
With the dishwasher	37 (37.8%)
Missing	7 (7.1%)
If you are using a dishwasher, take a preliminary step	
No	9 (9.2%)
Yes with paper	19 (19.4%)
Yes with brush	10 (10.2%)
Yes with sponge	25 (25.5%)
Missing	35 (35.7%)
How many family members use the sponge	
>2	20 (20.4%)
1–2	67 (68.4%)
Also domestic helper	4 (4.1%)
Missing	7 (7.1%)
How frequently do you use the sponge	
Occasionally	6 (6.1%)
Every day	83 (84.7%)
Missing	9 (9.2%)
Generally, when you replace the sponge	
Fixed periodicity	25 (25.5%)
Presence of cuts	37 (37.8%)
Dirty-smelly	20 (20.4%)
Missing	16 (16.3%)

Absolute and relative frequencies of demographic characteristics and household hygiene practices collected through the questionnaire, including presence of vulnerable individuals, age class, sex, sponge storage location, dishwashing practices, sponge usage frequency, and sponge replacement criteria (N = 98).

**Table 4:** Hygiene-related questionnaire variables.

Questionnaire 2	Overall (*N* = 98)
Specific knowledge of food safety	
No	29 (29.6%)
Yes	69 (70.4%)
Time in use of the sponge	
Less than 1 week	11 (11.2%)
More than 1 week	87 (88.8%)
Sanitize the sponge periodically	
No	32 (32.7%)
Yes	46 (46.9%)
Missing	20 (20.4%)

Absolute and relative frequencies of selected hygiene-related variables, including food safety knowledge, duration of sponge use, and periodic sponge sanitisation practices among the study participants (N = 98). Missing responses were reported separately and excluded from inferential analyses.

**Table 5:** Association between sponge score and hygiene-related variables.

Questionnaire 3	Acceptable (*N* = 48)	To be replaced (*N* = 50)	*P*-value
Specific knowledge of food safety			
No	13 (27.1%)	16 (32.0%)	0.661
Yes	35 (72.9%)	34 (68.0%)	
Time in use of the sponge			
Less than 1 week	7 (14.6%)	4 (8.0%)	0.352
More than 1 week	41 (85.4%)	46 (92.0%)	
Sanitize the sponge periodically			
No	12 (25%)	20 (40%)	0.06
Yes	28 (58.3%)	18 (36.0%)	
Missing	8 (16.7%)	12 (24%)	

Association between sponge condition (acceptable vs. to be replaced) and selected hygiene-related variables, including food safety knowledge, duration of sponge use, and periodic sponge sanitisation. Statistical significance was assessed using Fisher's exact test after exclusion of missing responses. Odds ratios were calculated to estimate the strength of associations. Missing or non-answer responses were excluded from inferential analyses.

**Table 6:** Association between sponge score and sponge sanitisation methods.

Questionnaire 4	Acceptable (*N* = 48)	To be replaced (*N* = 50)	*P*-value
Sponge cleaning			
boiling water	4 (8.3%)	2 (4.0%)	1
boiling water/disinfectant	2 (4.2%)	1 (2.0%)	
Disinfectants	21 (43.8%)	15 (30.0%)	
Dishwasher	1 (2.1%)	0 (0%)	
Microwave	1 (2.1%)	1 (2.0%)	
Missing	19 (39.6%)	31 (62.0%)	

Association between sponge condition (acceptable vs. to be replaced) and reported sponge sanitisation methods (boiling water, disinfectants, dishwasher, microwave). Statistical significance was assessed using Fisher's exact test.

### 3.2 *Pseudomonas* colony identification by 16S rDNA sequencing

**Figure 2:**
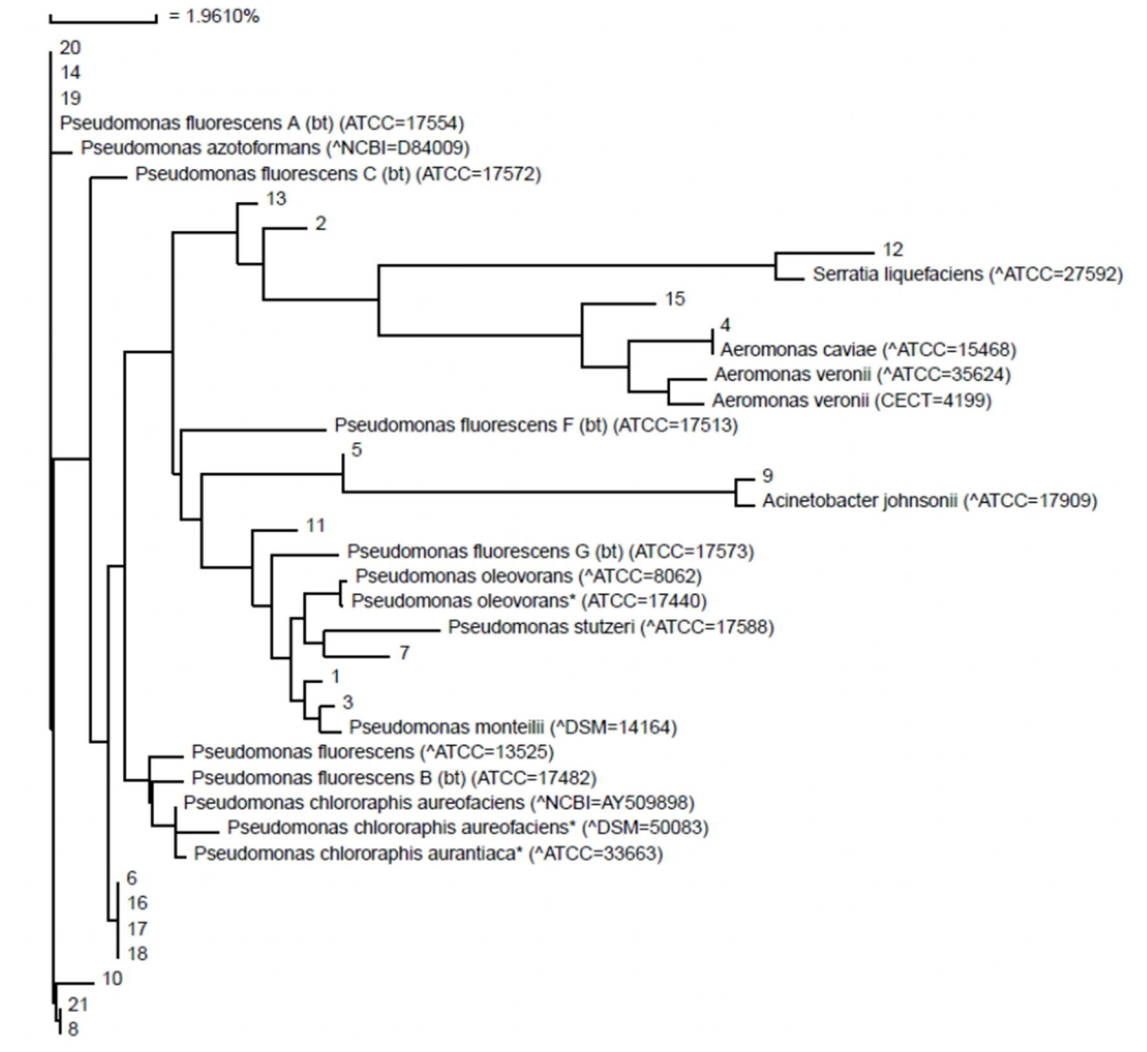
Phylogenetic tree based on partial 16S rRNA gene sequences obtained using the MicroSeq™ 16S protocol from 21 Pseudomonas isolates recovered from kitchen sponges within the 1-3 log CFU/sponge contamination category, together with selected reference strains. Phylogenetic relationships were inferred using (Neighbour-Joining/UPGMA). Branch lengths indicate genetic distance, and the scale bar represents nucleotide substitutions per site.

Partial 16S rRNA gene sequencing (MicroSeq™ 16S protocol) was successfully performed for all 21 isolates belonging to the *Pseudomonas* category within the “1–3 log” plate count range. Isolates were selected from this lower microbial load category, as samples with higher counts inherently represent an established microbiological concern. This approach was undertaken to assess whether clinically or environmentally relevant *Pseudomonas* species were already detectable at lower contamination levels and to characterize their phylogenetic relationships within the genus. High-quality sequences allowed reliable taxonomic assignment at species level based on database comparison. The isolates were were identified as shows in Table 7. The construction of the phylogenetic tree was performed on the Applied Biosystems MicroSEQ software^®^ ID Microbial Identification Software (Figure 2) and the phylogenetic relationships were inferred using both Neighbor-Joining (NJ) and UPGMA algorithms through the software BioEdit (Tom Hall, Ibis BioSciences). The NJ tree was constructed without enforcing ultrametricity, whereas UPGMA assumed a molecular clock. Overall, both methods produced largely congruent clustering patterns. The NJ tree revealed three principal clusters. Cluster I (*Pseudomonas monteilii* group Isolates 1, 3, and 11 clustered tightly together with very short branch lengths, indicating minimal intraspecific divergence. This grouping was stable across both NJ and UPGMA analyses. The short genetic distances suggest high sequence similarity within this taxon. Cluster II—*Pseudomonas fluorescens* complex and related species Isolates 6, 8, 14,16,17,18,19,20,21 (all identified as *P. fluorescens*) formed a coherent subcluster. The close association of isolates 6 and 8 was particularly evident, with very short branch lengths in both phylogenetic reconstructions. Isolates 7 (*P. stutzeri*) and 10 (*P. azotoformans*) grouped within the broader *Pseudomonas* clade but formed distinct sub-branches, reflecting interspecific divergence within the genus. Isolate 13 (*P. chlororaphis*) appeared more divergent within the *Pseudomonas* cluster, exhibiting a comparatively long branch length. Cluster III—Non-*Pseudomonas* taxa Isolates identified as *Aeromonas* spp. (2, 4, 15), *Acinetobacter johnsonii* (9), and *Serratia liquefaciens* (12) formed clearly separated lineages. Notably, 4 and 15 (both *Aeromonas caviae*) did not cluster as tightly as the *Pseudomonas monteilii* or *P. fluorescens* isolates, suggesting detectable intraspecific variation. 9 (*Acinetobacter johnsonii*) and 13 (*Pseudomonas chlororaphis*) displayed relatively long branches, indicating higher genetic divergence compared to the core *Pseudomonas* clusters. 12 (*Serratia liquefaciens*) formed a distinct lineage separate from both *Pseudomonas* and *Aeromonas* clusters. The UPGMA tree, constructed under the assumption of rate constancy, produced an ultrametric topology consistent with the major groupings observed in the NJ tree. In particular: The tight clustering of *P. monteilii* isolates (1, 3, 11) was confirmed the close association between *P. fluorescens* isolates (6, 8, 14,16,17,18,19,20,21) was maintained, the separation of non-*Pseudomonas* genera (*Aeromonas, Acinetobacter, Serratia*) from the main *Pseudomonas* cluster was clearly supported. Although branch lengths differed between methods, no major topological incongruences were observed at the genus level.

**Table 7:** Phylogenetic affiliation of isolates based on 16S rRNA gene sequencing.

Isolate	Species	Phylogenetic group within Pseudomonas	Ecological traits
1, 3, 11	*P. monteilii*	P. fluorescens group	Environmental, siderophore-producing
6, 8, 14, 16,17,18,19,20,21,	*P. fluorescens*	P. fluorescens group	Fluorescent siderophores, biofilm-forming
10	*P. azotoformans*	P. fluorescens group	Nitrogen metabolism, environmental
13	*P. chlororaphis*	P. fluorescens group	Secondary metabolites, biocontrol traits
5	*P. oleovorans*	P. putida group	Hydrocarbon degradation
7	*P. stutzeri*	P. stutzeri group	Denitrification, genomic plasticity
2	*Aeromonas veronii*	Aeromonadaceae	Aquatic-associated, opportunistic
4, 15	*Aeromonas caviae*	Aeromonadaceae	Aquatic, opportunistic
9	*Acinetobacter johnsonii*	Moraxellaceae	Environmental, stress tolerant
12	*Serratia liquefaciens*	Enterobacterales	Environmental, AmpC producer

## 4 Discussion

This study provides a preliminary overview of microbial contamination patterns and hygiene-related behaviors associated with domestic kitchen sponge use. First, no *a priori* sample-size calculation was performed, as the analyzed data derived from a broader exploratory research project. Although the cross-sectional design does not allow causal inference, the findings offer useful insights into associations between sponge maintenance practices and sponge condition in real household settings. The culture-based microbiological approach enabled the identification of cultivable microorganisms commonly associated with domestic wet environments, while partial 16S rRNA sequencing provided an initial descriptive characterisation of selected isolates. Nevertheless, the microbiological findings represent only the cultivable fraction of the sponge-associated microbiota, and comprehensive microbiome profiling was beyond the scope of the present study. Future investigations using longitudinal designs and amplicon-based or metagenomic approaches could provide broader insights into microbial community composition, functional traits, and the potential impact of hygiene practices over time. Due to the limited sample size and the distribution of some questionnaire variables, no multivariable model was applied; therefore, potential confounding factors cannot be completely excluded. Finally, the sponge scoring system was based on operator assessment and should be further standardized and validated in future studies. The present study suggests that kitchen sponges may harbor heterogeneous microbial populations within the domestic environment. The extremely high microbial loads observed in some samples, particularly for Enterobacteriaceae and *Pseudomonas* spp., are consistent with previous studies describing sponges as environments favorable to microbial persistence and growth due to their porosity, moisture retention, and frequent contact with food residues (Cardinale et al., 2017; Jacksch et al., 2020). The dominance of Gram-negative bacteria, especially Enterobacteriaceae and *Pseudomonas* spp., is of particular concern, as these groups include opportunistic pathogens and spoilage organisms capable of forming resilient biofilms (Coughlan et al., 2016; Møretrø et al., 2022a). The frequent detection of yeasts and molds further supports the notion that kitchen sponges promote the survival of a broad range of microorganisms, including those involved in food spoilage and allergic responses. Using 16S rRNA gene sequencing, (Cardinale et al., 2017) demonstrated that kitchen sponges can harbor highly diverse bacterial communities, including Gammaproteobacteria such as Pseudomonas, Acinetobacter, and members of the Enterobacteriaceae. These observations are consistent with the microorganisms identified in the present study and support previous evidence that domestic kitchen sponges may sustain diverse microbial populations. At the same time, culture-based enumeration methods may underestimate the overall microbial diversity associated with these environments (Amirfard et al., 2024). Accordingly, the microbiological findings presented here should be interpreted as representing primarily the cultivable fraction of the sponge-associated microbiota. Partial 16S rRNA gene sequencing revealed that most isolates recovered from kitchen sponges belonged to the *Pseudomonas fluorescens* species group, including *P. fluorescens, P. chlororaphis, P. monteilii* and *P. azotoformans*. that are well recognized environmental generalists adapted to moist, nutrient-variable habitats (Chen et al., 2024). Kitchen sponges represent a highly hydrated microenvironment enriched in organic residues, fluctuating temperatures and periodic exposure to detergents and disinfectants, conditions that strongly favor metabolically versatile Gammaproteobacteria (Esteves et al., 2017). A central finding of this study is the absence of statistically significant associations between sponge condition, as assessed by visual and functional criteria, and microbiological contamination levels. Importantly, this result should not be interpreted as evidence of the absence of a biological relationship between sponge deterioration and microbial growth. Rather, it indicates that visual assessment alone does not reliably reflect microbiological status within the constraints of the present study. The absence of statistically significant associations in this study should be interpreted as a lack of detectable evidence rather than evidence of absence, particularly given the cross-sectional design, the categorical nature of the variables, and the inherent variability of domestic hygiene practices. The detection of substantial microbial loads in sponges classified as acceptable suggests that sensory cues such as appearance, texture, or odor may underestimate hygienic risk in domestic environments. This observation is consistent with previous studies demonstrating that microorganisms can persist deep within the sponge matrix, forming biofilms that are not readily detectable through superficial inspection (Kusumaningrum et al., 2003; Scott et al., 1982). From a practical perspective, these findings reinforce concerns regarding the use of visual criteria as proxies for microbiological safety. Previous studies of metagenomic analyses further indicate that microbial communities persist even after cleaning interventions, often undergoing compositional shifts rather than complete removal (Jacksch et al., 2020). Although the estimated odds ratio suggested a possible association between periodic sponge sanitisation and improved macroscopic sponge condition, this association did not remain statistically significant after revision of the missing-data handling strategy. Therefore, these findings should be interpreted cautiously and considered exploratory rather than confirmatory. Furthermore, due to the cross-sectional design of the study, the observed associations should not be interpreted as evidence of causality. However, no significant association was observed between sponge condition and the microbiological categories investigated. Previous experimental studies have shown that microwave treatment, boiling, and chemical disinfectants can reduce bacterial loads under controlled conditions Brandau et al., 2022; Sharma et al., 2009). However, the present cross-sectional study was not designed to evaluate the effectiveness of specific sanitisation procedures on microbial contamination levels. From an ecological perspective, sanitisation may selectively reduce sensitive taxa while allowing resilient organisms, such as *Pseudomonas* spp., to dominate post-treatment communities (Jacksch et al., 2020). No specific sanitisation method emerged as superior, which may reflect variability in execution, frequency, or duration of treatment. This aligns with previous work showing that while methods such as boiling, microwave treatment, and chemical disinfectants can be effective, their real-world efficacy depends heavily on user compliance and consistency (Borrusso and Quinlan, 2017). The absence of a significant association between food safety knowledge and sponge condition highlights a well-documented gap between knowledge and behavior in domestic food hygiene. Several studies have shown that awareness of food safety principles does not necessarily translate into safer practices, particularly in non-professional settings (Byrd-Bredbenner et al., 2013; Calderón-Franco et al., 2020; Redmond and Griffith, 2009) the knowledge alone rarely translates into sustained behavioral change. This disconnect may be exacerbated by the perception of the home as a “safe” environment, in contrast to professional food-handling settings. In domestic settings, hygiene practices are often influenced not only by knowledge, but also by routine habits, perceived convenience, time constraints, and subjective perceptions of cleanliness. In addition, behavioral variability among households may be affected by demographic or socio-cultural factors that were not specifically investigated in the present study. Although the current dataset was not designed for stratified behavioral analysis, the frequent presence of vulnerable individuals in participating households highlights the importance of further investigating how household composition may influence domestic hygiene practices and risk perception (Du et al., 2015). This finding suggests that educational interventions alone may be insufficient and that practical guidance or behavioral-oriented strategies, such as clearer recommendations regarding sponge replacement and sanitisation practices, could support improved domestic hygiene behaviors (Carstens et al., 2024). In addition, the frequent presence of vulnerable individuals in participating households highlights the importance of maintaining adequate kitchen hygiene practices, as exposure to opportunistic microorganisms may represent a potential concern for elderly individuals, young children, pregnant women, and immunocompromised persons (Bloomfield, 2001; Carstens et al., 2022).

## 5 Conclusions

This study demonstrates that domestic kitchen sponges frequently harbor high and highly variable microbial loads, confirming their role as important reservoirs of microorganisms within the household environment. The absence of a clear relationship between visual sponge condition and microbiological contamination highlights the limitations of sensory-based assessments and reinforces the concept that apparent cleanliness does not equate to microbiological safety. Periodic sponge sanitization showed a possible association with improved macroscopic sponge condition; however, this association was not statistically robust. These findings suggest that visual appearance alone may not represent a reliable indicator of microbiological status. Further studies are needed to determine whether domestic sponge sanitisation practices effectively reduce microbial contamination and food-safety risk. Taken together, the results underscore the need for practical, behavior-focused public health guidance addressing sponge hygiene and replacement, particularly in households with vulnerable individuals. Recognizing that kitchen sponges may harbor diverse microorganisms under routine domestic use conditions rather than inert cleaning tools may support more effective domestic hygiene strategies and may contribute to reducing potential microbial exposure in everyday food-handling environments.

## Data Availability

The data presented in the study are deposited in the GenBank repository, accession number PZ520532-PZ520551.
